# Validity and reliability of a wearable armband for continuous sweat conductivity monitoring during exercise

**DOI:** 10.3389/fphys.2026.1809119

**Published:** 2026-04-30

**Authors:** Antoine Ferrari, Sébastien Ratel, Serge Berthoin, Jean-Philippe Garnier, Georges Baquet

**Affiliations:** 1 Univ. Lille, Univ. Artois, Univ. Littoral Côte d’Opale, Unité de Recherche Labellisée (ULR) n°7369 - Unité de Recherche Pluridisciplinaire Sport Santé Société (URePSSS), Lille, France; 2 Clermont-Auvergne University, Laboratoire des Adaptations Métaboliques è l’Exercice en conditions Physiologiques et Pathologiques (AME2P), Clermont-Ferrand, France; 3 Centre d’Investigation Clinique – Innovation Technologique (CIC-IT) (Institut National de la Santé et de la Recherche Médicale (INSERM) 1403), Centre Hospitalier Universitaire de Lille, Lille, France

**Keywords:** conductivity, electrolytes, hydration, microfluidics, sweat testing, thermoregulation

## Abstract

**Introduction:**

Sweating is essential for thermoregulation during exercise but causes variable losses of water and sodium chloride (NaCl). Field-based methods to assess these losses are limited by sampling errors and the lack of real-time feedback. This study evaluated the validity and reliability of the BeOne armband for continuous measurement of sweat conductivity, used to estimate sweat [NaCl] equivalent in real time.

**Methods:**

Ten armbands were first tested against standard NaCl solutions (5–120 mmol·L^−1^) to assess the validity of the conductivity measurement. Reliability was then examined in sixteen men who completed two 60-min cycling sessions at 150 W in temperate condition (24 ± 1 °C; 50 ± 10% RH) while wearing armbands on both forearms. Outcomes included sweat onset (defined as the time from exercise onset to first detection of sweat conductivity), sweat [NaCl] equivalent drift, and time-normalized [NaCl] equivalent kinetics.

**Results:**

Measured values showed minimal bias (0.28 mmol·L^−1^, 95% LoA: –1.21 to 1.77), very low CVs (0.5–2.6%), and excellent inter-device agreement (ICC = 1.00) for conductivity measurements. During exercise, sweat onset did not differ between arms or sessions, but showed moderate within-session (ICC = 0.72) and low between-session reliability (ICC = 0.29), indicating substantial day-to-day variability of this specific index. Drift indices and time-normalized [NaCl] equivalent kinetics revealed a reproducible temporal profile, characterized by an initial peak, a rapid decline, a gradual increase, and a plateau toward the end of exercise.

**Conclusion:**

The BeOne armband demonstrated excellent analytical validity for sweat conductivity measurement and reliable within-session characterization of conductivity-derived sweat electrolyte dynamics. Although conductivity-based [NaCl] equivalents do not represent a laboratory gold standard and remain sensitive to matrix effects and non-specific ionic contributions, this approach appears sufficiently robust for applied sport-science contexts requiring continuous monitoring.

## 1 Introduction

Evaporative heat loss through sweating is a key mechanism of human thermoregulation during exercise (Gagge and Nishi, 1977). However, sweat production also results in substantial fluid and electrolyte losses, particularly sodium and chloride (Baker, 2017, 2019). These losses are highly variable, both between and within individuals (Baker, 2017), depending on factors such as exercise intensity (Buono et al., 2008), environmental conditions (Dziedzic et al., 2014), heat acclimation status (Buono et al., 2007), and dietary sodium intake (Allsopp et al., 1998). In prolonged or intense endurance exercises, particularly in hot and humid environments, excessive sweating can cause marked disturbances in fluid and sodium balances, which may impair performance (Racinais et al., 2015) and increase the risk of dehydration and hyponatremia (Armstrong, 2021; Knechtle et al., 2019). Accordingly, individualized hydration strategies that consider both water and sodium replacements are recommended for athletes (Armstrong, 2021; McCubbin et al., 2020).

Whole-body sweat rate (WBSR) can be estimated by measuring pre- to post-exercise body mass changes (Baker, 2017). In contrast, quantifying electrolyte losses through sweat remains more challenging. The reference technique, the whole-body washdown method, involves rinsing and collecting all sweat produced during exercise for laboratory analysis (Shirreffs and Maughan, 1997). While accurate, this procedure is time-consuming, requires specialized facilities, and is impractical in field settings (Baker et al., 2009). Alternative approaches, such as absorbent patches or skin-applied microfluidic devices, allow local sweat collection for subsequent laboratory analysis, which is then used to predict whole-body electrolyte losses (Baker et al., 2009; Patterson et al., 2000). Although these methods are widely used in both research and applied contexts, they remain prone to sampling errors. Patches cannot be worn for extended periods without risk of detachment, saturation, or contamination and should ideally not be applied at the very onset of sweating to avoid initial contamination (Baker, 2017). Furthermore, subsequent analyses require costly laboratory equipment (ion chromatography, flame photometry, direct and indirect ion selective electrode), and because these techniques provide only discrete time-point measurements, they offer limited value for real-time hydration monitoring and decision-making during exercise.

Recent advances in wearable biosensor technology have transformed the assessment of sweat composition, enabling continuous and non-invasive measurements directly on the skin (Hashimoto et al., 2022; Pirovano et al., 2020; Relf et al., 2020; Wang et al., 2023). This rapidly expanding field also includes flexible potentiometric and hybrid electrochemical platforms specifically developed for ion-selective sweat sensing, highlighting the growing technological diversity of real-time wearable monitoring systems (Fathy and Bühlmann, 2025). Unlike traditional patch-based methods that rely on discrete sampling and laboratory analysis, these systems provide real-time data acquisition, offering valuable insight into the temporal dynamics of sweat electrolyte responses during exercise. Continuous monitoring represents a major step forward, particularly for athletes, as it allows instant feedback on hydration status and salt loss trends under real-world conditions. Several wearable systems have recently been proposed to address these challenges, including patch-based microfluidic devices (e.g., Gx Sweat Patch; Baker et al., 2022a) and emerging continuous sensors designed for field use (e.g., Flowbio; Bandiera et al., 2026), highlighting the growing interest in real-time sweat sensing. In this evolving landscape, the BeOne armband represents an additional solution based on continuous sweat conductimetry, whose performance requires dedicated evaluation. Rather than targeting laboratory-grade quantification of individual ions, the BeOne is designed to continuously measure sweat conductivity and derive a sweat [NaCl] equivalent (Boisvert and Candas, 1994; Goulet et al., 2017), enabling uninterrupted tracking of intra-session sweat electrolyte dynamics throughout exercise. As with other conductivity-based wearable systems, this approach does not provide ion-specific quantification and may be influenced by the overall ionic composition of sweat. However, it currently remains one of the most feasible solutions for continuous field-based monitoring. Its lightweight, easy-to-use design and connection to a smartphone application enable real-time visualization of the data, facilitating both scientific interpretation and immediate application for athletes and practitioners.

To ensure the scientific robustness of this new monitoring technology, it is essential to evaluate both its analytical validity for conductivity measurement and its measurement reliability under controlled and practical conditions. Therefore, the present study aimed to assess the analytical validity of conductivity measurement and the *in vivo* reliability of conductivity-derived sweat [NaCl] equivalent monitoring during exercise. The investigation was divided into two complementary phases. Part A evaluated the analytical validity of the device using standardized NaCl solutions of known concentrations, specifically to verify the instrument’s ability to accurately measure the conductivity of a solution, i.e., the fundamental signal from which the device derives its [NaCl] estimates. This step is essential to confirm the accuracy and consistency of the BeOne’s sensing principle and to assess inter-device agreement across units. Part B focused on *in vivo* reliability, with Part B.1 examining possible side-related effects by comparing simultaneous recordings from the left and right forearms, and Part B.2 assessing between-session reproducibility through a test–retest design. Together, these analyses provide a comprehensive framework for evaluating the BeOne system as a reliable and field-ready tool for monitoring sweat electrolyte dynamics.

## 2 Materials and methods

### 2.1 Study design

The present investigation was designed to evaluate the validity and reliability of the BeOne Connected Armband, a wearable device providing continuous monitoring of sweat [NaCl] equivalent. Given the continuous nature of the measurement, the experimental protocol was structured in two complementary phases.

In Part A, the analytical validity of the device was assessed under controlled laboratory conditions. Ten armbands were exposed to standard [NaCl] solutions spanning a physiological range of concentrations, enabling evaluation of measurement accuracy and inter-device variability.

In Part B, the reliability of the device was examined during exercise. Sixteen men performed two standardized cycling sessions while wearing armbands on both forearms. This protocol allowed assessment of the device’s capacity to monitor dynamic changes in conductivity-derived sweat [NaCl] equivalent *in vivo*, as well as its reproducibility under repeated testing conditions. Part B.1 examined the side effect by comparing right and left arm measurements, and Part B.2 investigated the session effect through a test–retest design.

### 2.2 Experimental procedure

#### 2.2.1 Part A: standard solutions

Ten BeOne armbands were tested using [NaCl] solutions at predefined concentrations (5, 10, 20, 30, 50, 80, and 120 mmol·L^−1^). This range was selected to reflect the physiological spectrum of human sweat sodium concentrations ([Na^+^]), typically reported between 10 and 90 mmol·L^−1^ (Baker and Wolfe, 2020). In the present study, the BeOne did not directly assess sweat [Na^+^] but rather a [NaCl] equivalent derived from solution conductivity. Accordingly, Part A was designed to evaluate the analytical performance and inter-device agreement of the conductivity-based measurement under controlled ionic conditions, rather than to establish physiological equivalence with laboratory-based sweat sodium analyses. Thus, the aim of this phase was not to reproduce the biochemical complexity of human sweat, but to assess the analytical precision, linearity, and inter-device validity of the conductimetric signal under standardized ionic conditions. Using NaCl standards is a common and appropriate approach for validating conductivity-based sensors, as Na^+^ and Cl^−^ account for the vast majority of sweat ionic strength and thus largely determine its conductive behavior (Baker and Wolfe, 2020). Previous comparisons between conductivity-based NaCl estimates and ion-specific [Na^+^] analyses have reported higher values for [NaCl] (i.e., ~12–15% higher than flame photometry; Boisvert and Candas, 1994; Goulet et al., 2017). For this reason, calibration solutions extended to 120 mmol·L^−1^ were included to encompass the full range expected when measuring sweat conductivity.

Stock [NaCl] solution (2000 mmol·L^−1^) was diluted with deionized water to the desired concentrations, and the final values were verified using a Sweat-Check™ NaCl analyzer (Elitech Group, Logan, UT, USA). This instrument is widely used in clinical settings, including cystic fibrosis diagnostics, making it a suitable referencefor a conductivity-based device (Hammond et al., 1994; Mastella et al., 2000). The Sweat-Chek was used here as a reference conductivity analyzer under controlled laboratory conditions to verify the conductimetric response of the BeOne, rather than as an ion-specific reference for sweat sodium quantification. Calibration and verification procedures for the reference measurements followed the manufacturer’s recommended protocol and were performed under controlled laboratory temperature conditions to ensure measurement consistency. For laboratory testing, aliquots of each standard solution were directly introduced into the Sweat-Chek according to the manufacturer’s instructions for liquid sample analysis. For each concentration, a dedicated microfluidic collector was filled with the solution, and measurements were obtained sequentially with all ten armbands. For each device, data were recorded for 10 seconds per concentration, and the mean value over this period was used for analysis. This approach ensured that each device was tested under identical conditions for each concentration.

#### 2.2.2 Part B: test–retest in athletes

Participants: Sixteen men volunteered to participate in this study (age: 26.5 ± 3.5 y; height: 178.5 ± 5.9 cm; body mass: 82.5 ± 16.0 kg). Only male participants were included in the present study in order to minimize potential variability in thermoregulatory and sudomotor responses related to menstrual cycle phase and associated hormonal fluctuations, which are known to influence sweating responses and sweat electrolyte composition (Inoue et al., 2005; Janse De Jonge et al., 2012; Kolka and Stephenson, 1989). This methodological choice was intended to strengthen the internal validity of the device validation and reliability analyses under controlled conditions. The study took place between April and July 2024 (i.e., northern hemisphere spring in Lille, France); participants were therefore assumed to be non-heat acclimatized and were instructed to avoid deliberate heat exposure between sessions. The required sample size was determined *a priori* based on the detection of a medium effect size (d = 0.4) for the primary repeated-measures comparison of sweat onset, with statistical power set at 80% and α = 0.05. This calculation indicated that 16 participants were sufficient, and recruitment continued until this number was reached. Because no prior data were available for continuous conductivity-derived variables obtained with this novel device, a precision-based *a priori* sample size calculation for reliability outcomes (e.g., ICC estimation) could not be performed. To be included, the participants were between 18 and 35 years old, practicing a minimum of 3 hours of endurance sport or team sport per week and were free of any injury or illness. This study was approved by a local institutional ethics review board (Ethics committee for research, Lille University, n°2023-665-S113). The study was conducted in conformity with the policy statement regarding the use of human subjects by the Declaration of Helsinki (2024, 75**^e^** General Assembly of the WMA). All experimental procedures were clearly explained to the participants, who then gave written consent prior to testing.

Participants refrained from strenuous exercise and alcohol consumption for at least 24 h before testing and were asked to maintain habitual dietary practices. To ensure euhydration, participants were instructed to consume 6 mL·kg^−1^ of body mass of water over the 2–3 h preceding each session.

Each participant completed two experimental sessions, separated by 2–7 days, and performed at the same time of day for that individual to minimize circadian influences. Upon arrival at the laboratory, a urine sample was collected for the assessment of urine specific gravity (USG; PEN-Urine S.G., ATAGO, Tokyo, Japan). When USG exceeded 1.020, participants ingested 5 mL·kg^−1^ of body mass of water within the 30 min preceding exercise. Nude body mass (underwear only) was then recorded using a calibrated platform scale (BodPod, Cosmed S.r.l, Rome, Italy).

Before exercise, participants were fitted with a heart rate monitor (Polar Electro Oy, Kempele, Finland) and two BeOne armbands, one on each forearm, positioned 8 cm below the cubital crease on the ventral aspect of the forearm. A new microfluidic collector was inserted into each device, and the forearm skin was cleaned with deionized water prior to placement to minimize contamination from residual electrolytes (Ely et al., 2011).

The exercise protocol consisted of 60 min of continuous cycling at 150 W, performed in a semi-controlled environment (24 ± 1 °C; 50 ± 10% relative humidity). This workload and duration were selected to ensure that all participants meeting the inclusion criteria could complete the session while eliciting sufficient and sustained sweating for reliable data collection. Ratings of perceived exertion (RPE; Borg, 1982) were obtained at 15, 30, 45, and 60 min during continuous exercise using the 6–20 Borg scale, on which participants rated their subjective perception of effort, ranging from “no exertion at all” (6) to “maximal exertion” (20). Post-exercise nude body mass was re-evaluated to estimate WBSR. No fluid intake was permitted during the trial.

### 2.3 BeOne testing system

**Figure 1:**
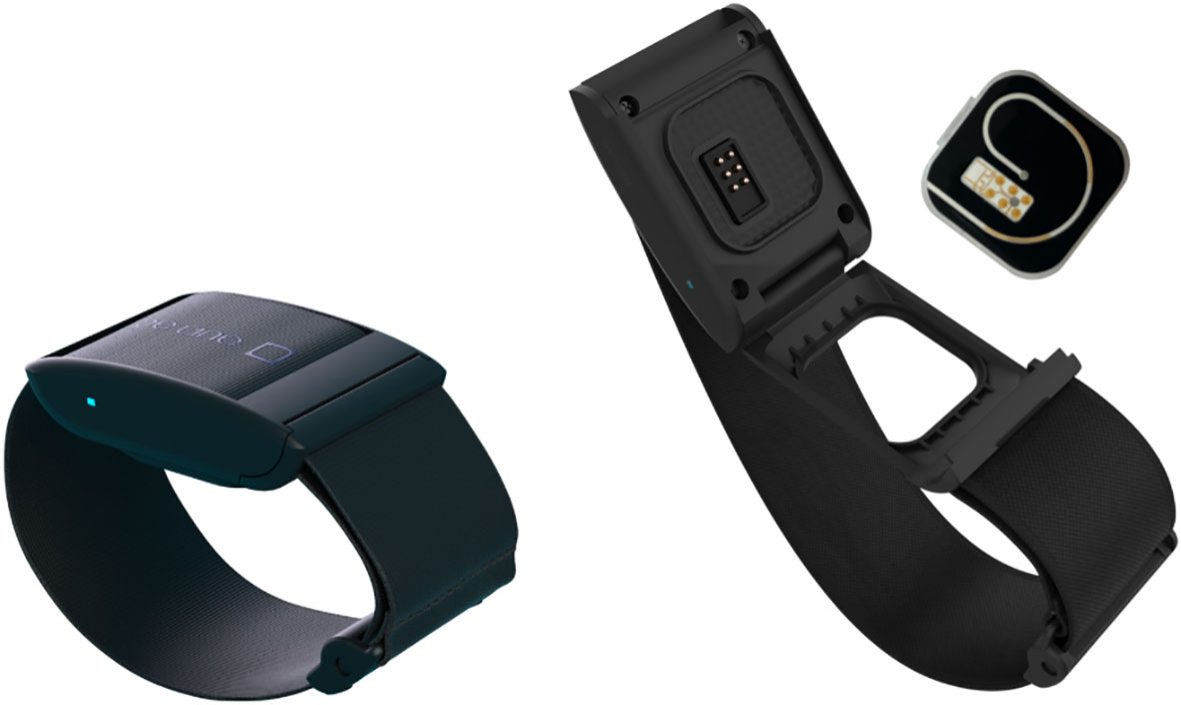
BeOne connected armband. Closed configuration (left) and open view showing the microfluidic collector (right).

The BeOne Connected Armband is a wearable system designed for continuous monitoring of sweat conductivity (**Figure 1**). The device integrates a sweat collector coupled with a coiled microfluidic channel that guides the sweat sample from the skin surface through the sensing area, allowing uninterrupted measurement throughout exercise.

The armband (~35 g, 180 mm × 45 mm × 15 mm) was worn on the ventral aspect of the forearm, a site chosen because it provides easy access during exercise and is commonly used in sweat studies to predict whole-body [Na^+^] losses (Patterson et al., 2000). The device interfaced with a 6.25 cm^2^ skin surface. Sweat entering the central orifice of the collector was directed through the microchannel (6.5 cm in length, 1.1 × 0.127 mm cross-section), where a pair of gold (Au) curvilinear electrodes (0.2 × 0.2 × 10 mm) continuously measured the electrical properties of the sweat as it flowed through the channel.

An alternating voltage signal (200 kHz, 215 mV) was applied across the sensing area using a voltage divider circuit to minimize electrode interface effects and estimate the bulk conductivity of the sweat solution. The excitation frequency was selected based on preliminary bench testing across the target concentration range, which demonstrated stable linearity and signal sensitivity within the expected physiological sweat range. The resulting voltage variation across the electrodes was used as the primary variable for estimating sweat [NaCl] equivalent. Measurements were sampled at 100 Hz, averaged every second, and transmitted via Bluetooth to a dedicated smartphone application for real-time visualization.

### 2.4 BeOne armband outcomes

**Figure 2:**
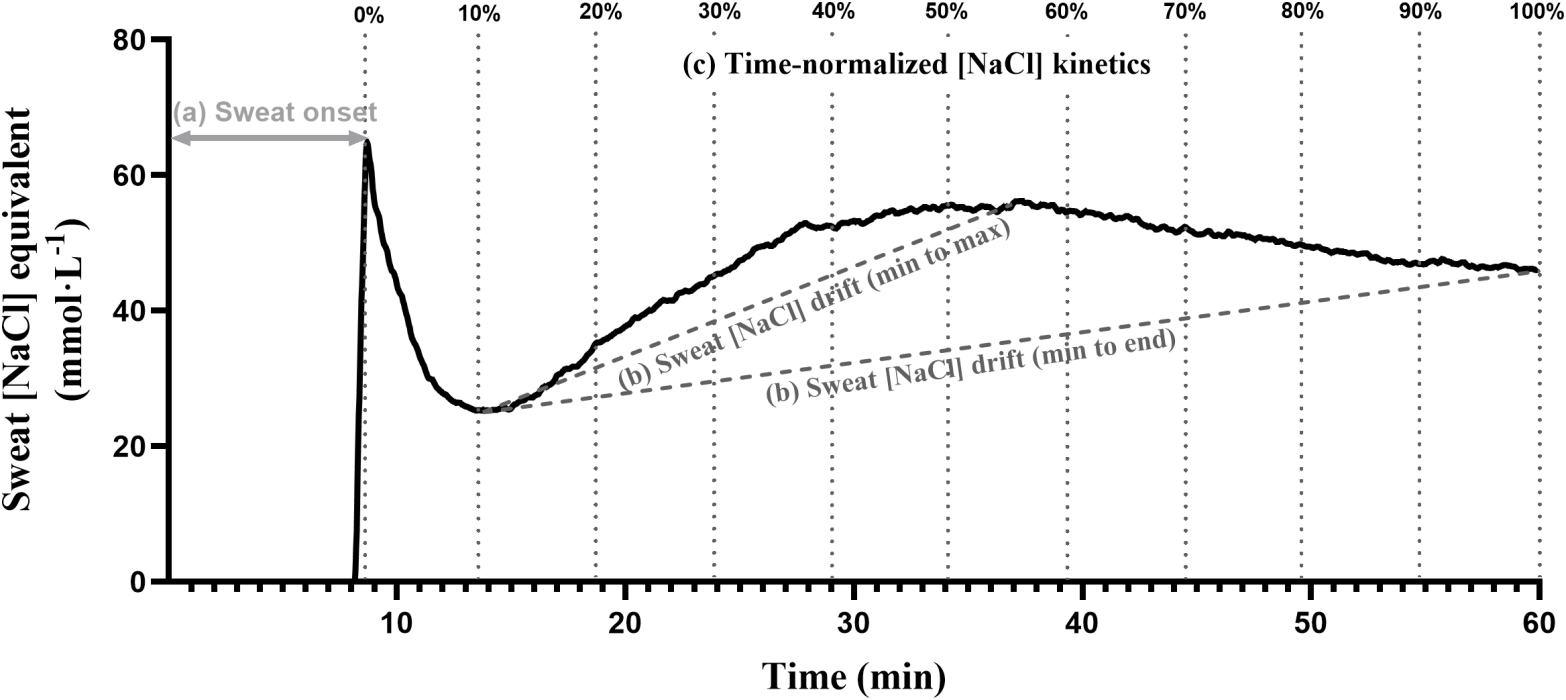
Example of a continuous sweat [NaCl] equivalent recording from the BeOne armband. Representative trace illustrating the three main outcome variables: (1) sweat onset (time from exercise start to initial detection), (2) sweat [NaCl] equivalent drift following the initial peak (min–max or min–end), and (3) time-normalized [NaCl] equivalent kinetics across the full exercise duration (0–100%).

Continuous recordings from the BeOne armband provided one measurement per second, enabling detailed visualization of individual sweat [NaCl] equivalent profiles during exercise. As illustrated in **Figure 2**, each trace typically exhibited three distinct phases: (a) a delay corresponding to the time required for sweat to be produced and reach the electrodes, (b) a sharp initial peak in [NaCl] equivalent coinciding with the first detection of sweat, and (c) a subsequent decline followed by stabilization or a gradual rise over time. Although the magnitude and timing of these phases varied between participants, this representative example illustrates the three key analytical outcomes derived from the signal—sweat onset, sweat [NaCl] equivalent drift, and time-normalized [NaCl] equivalent kinetics—which are described below.

- Sweat onset: defined as the time elapsed from the start of exercise until the armband first detected sweat, corresponding to the combined delay of physiological sweat gland activation and sweat transport through the microfluidic channel to the first pair of electrodes. This delay reflects the physiological process by which rising core and skin temperatures during exercise activate eccrine sweat glands, initiating sweat secretion that progressively reaches the sensing area until detection occurs.
- Sweat [NaCl] equivalent drift: Following sweat onset, [NaCl] equivalent typically exhibited an initial peak, a subsequent decline, and either stabilization or a progressive increase. For each trial, three reference points were identified: the minimum (min), corresponding to the lowest 10-s moving average after the initial peak; the maximum (max), the highest 10-s average thereafter; and the end, the mean of the final 10 s of recording. Drift was defined as the change in [NaCl] equivalent following the initial peak and was quantified either as a relative change (%; min–max or min–end), reflecting the amplitude of variation, or as an absolute rate of change (mmol·L^−1^·min^−1^; min–max or min–end), accounting for the time over which this variation occurred. **Figure 2** illustrates an example of distinct min–max and min–end drifts, although in some cases [NaCl] equivalent rose continuously or plateaued rapidly after the decline.
- Time-normalized [NaCl] equivalent kinetics: because sweat onset varied across participants and sessions, sweat [NaCl] equivalent kinetics were normalized to the percentage of measurement duration (0–100%), where 0% corresponded to sweat onset and 100% to the end of exercise. To generate comparable profiles, raw data were averaged over 10-second windows at each normalized time point. For instance, 0% values corresponded to the mean of the first 10 s following sweat onset, 100% values to the last 10 s of the session, and intermediate values (e.g., 10%, 20%, …, 90%) to the mean of 10-s segments centered on the corresponding percentage of total measurement duration. This allowed direct comparison of concentration profiles across arms and sessions.

### 2.5 Statistics

Data are presented as mean ± standard deviation (SD). Normality of distributions was verified using the Shapiro–Wilk test.

Part A. The inter-device validity of the BeOne armband was evaluated across [NaCl] calibration solutions of known concentrations. Between-device variability at each concentration was quantified using the coefficient of variation (CV), with interpretive thresholds defined as very good (<10%), good (10–20%), acceptable (20–30%), or not acceptable (>30%) (Atkinson and Nevill, 1998). Overall inter-device agreement was further assessed using the intraclass correlation coefficient (ICC) calculated with a two-way random-effects model for absolute agreement on single measurements [ICC(2,1)]. ICC values were interpreted as follows: low (<0.50), moderate (0.50–0.75), good (0.75–0.90), and excellent (>0.90) (Koo and Li, 2016), and were presented with a 95% confidence interval (CI) to reflect estimate precision. Agreement across devices was examined using Bland–Altman analysis (mean bias and 95% limits of agreement (LoA)) (Bland and Altman, 1986). Linear associations between calibration values and device output were assessed using Pearson’s correlation coefficient (two-tailed).

Part B.1. For within-session comparisons (right vs. left arm), paired t-tests or Wilcoxon signed-rank tests were applied to sweat onset time and to drift variables, expressed either as percentage change from baseline or as absolute rate of change (mmol·L^−1^·min^−1^). The reliability of sweat onset was further assessed using an ICC reflecting consistency between repeated measurements within individuals, along with the standard error of measurement (SEM) and the minimal detectable change at the 95% confidence level (MDC95). Time-normalized [NaCl] equivalent kinetics were analyzed using repeated-measures ANOVA with factors time, side, and time × side. If the p value was significant, Tukey’s *post hoc* tests were applied.

Part B.2. For between-session comparisons (test–retest), the same statistical procedures as in Part B.1 were applied, with session replacing side as the repeated factor. Additionally, paired t-tests were used to compare WBSR, and repeated-measures ANOVA was used to evaluate heart rate and RPE across time points.

All analyses and figure generation were performed with Prism 10.5 (GraphPad Software Inc., La Jolla, CA, USA). The level of statistical significance was set at *p* < 0.05.

## 3 Results

### 3.1 Part A: analytical validity with standard solutions

**Figure 3:**
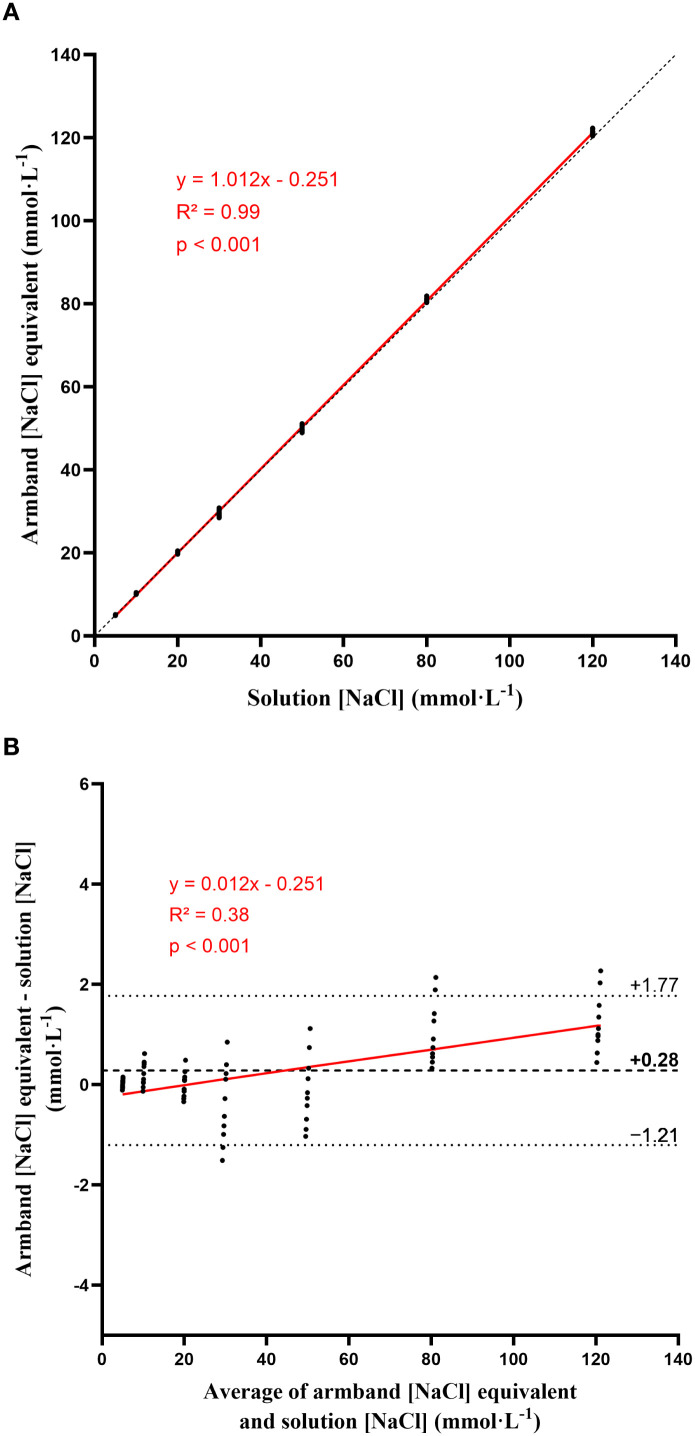
Correlation (red solid line) plot between the [NaCl] calibration solutions and measurement obtained from the BeOne armband (**A**); black dashed line represents the line of identity. Bland–Altman plot showing the mean difference (thick black dashed line) and the 95% limits of agreement (thin black dashed lines) between the BeOne measurements and the corresponding calibration solution values (**B**). The red solid line represents the linear regression of the differences against the mean concentrations used to assess proportional bias.

Detailed descriptive results are presented in **Table 1** and **Figures 3A, B**. Across the seven NaCl calibration solutions (5, 10, 20, 30, 50, 80, and 120 mmol·L^−1^), the mean bias was 0.28 mmol·L^−1^ with 95% LoA ranging from −1.21 to 1.77 mmol·L^−1^ (**Figure 3B**). Additional linear regression analysis of the Bland–Altman differences against the corresponding mean concentrations revealed a statistically significant proportional bias (slope = 0.0119, p < 0.001). CVs were consistently low, ranging from 0.48% at 120 mmol·L^−1^ to 2.61% at 30 mmol·L^−1^. The ICC reached 1.00 (95% CI: 0.99 to 1.00), indicating excellent inter-device agreement. Pearson’s correlation coefficient confirmed a nearly perfect linear association between calibration values and device output (r = 0.99, 95% CI 0.99–0.99, R^2^ = 0.99, p < 0.001). Overall, mean measured values closely matched the target conductivity-equivalent concentrations, with minimal variability between devices.

**Table 1:** Mean ± SD values and coefficients of variation (CV) for calibration solutions.

[NaCl] calibration solution (mmol·L^-1^)	BeOne measured [NaCl] equivalent (mmol·L^-1^, mean ± SD)	Inter-device CV(%) (95% CI)
5	5.02 ± 0.09	1.708 (0.919 - 2.498)
10	10.21 ± 0.24	2.391 (1.286 - 3.495)
20	20.00 ± 0.26	1.311 (0.705 - 1.916)
30	29.61 ± 0.77	2.614 (1.406 - 3.822)
50	49.89 ± 0.70	1.401 (0.754 - 2.048)
80	81.03 ± 0.62	0.770 (0.414 - 1.126)
120	121.23 ± 0.59	0.484 (0.260 - 0.708)

CI, confidence interval; CV, coefficient of variation; [NaCl], Sodium chloride concentration; SD, Standard Deviation.

### 3.2 Part B.1: side effect (right vs. left arm)

Sweat onset time did not significantly differ between arms (698 ± 215 s left vs. 664 ± 181 s right; p = 0.25). The reliability of sweat onset across arms was moderate, with ICC = 0.72 (95% CI: 0.50–0.85), corresponding to a SEM of 105 s and an MDC95 of 292 s. Percentage drifts did not significantly differ between arms for either the min–max (p = 0.26) or min–end (p = 0.11) comparisons. The absolute rate of change from minimum to maximum was higher on the left arm (0.63 ± 0.55 vs. 0.49 ± 0.38 mmol·L^−1^·min^−1^; p < 0.01), while the absolute drift from minimum to end-exercise did not significantly differ (p = 0.09). For time-normalized [NaCl] equivalent kinetics, repeated-measures ANOVA revealed no significant time × side interaction (*F*_(10,62)_ = 0.05, p > 0.99, η^2^ = 0.001). There was no effect of side (*F*_(1,62)_ = 0.002, p = 0.96, η^2^ = 0), indicating similar concentration profiles between arms. However, there was a significant effect of time (*F*_(10,62)_ = 41.3, p < 0.001, η^2^ = 0.4), reflecting systematic changes in sweat [NaCl] equivalent over the course of exercise.

### 3.3 Part B.2: session effect (test–retest)

Pre-exercise USG did not significantly differ between trials (session 1: 1.012 ± 0.012; session 2: 1.011 ± 0.007; p = 0.63). For both heart rate and RPE, repeated-measures ANOVA revealed a significant main effect of time, with values progressively increasing throughout exercise (heart rate: *F*_(3,69)_ = 140.9, p < 0.001, η^2^ = 0.83; RPE: *F*_(2,45)_ = 36.2, p < 0.001, η^2^ = 0.58), but no effect of session or time × session interaction (p > 0.1 and η^2^ < 0.07 for both). WBSR was also similar across sessions (0.97 ± 0.16 vs. 0.98 ± 0.15 L·h^−1^; p = 0.46). Overall, hydration status, physiological responses, and perceived exertion remained consistent between sessions. Sweat onset time did not significantly differ between sessions (687 ± 218 s session 1 vs. 675 ± 179 s session 2; p = 0.96). Test–retest reliability for sweat onset was low, with ICC = 0.29 (95% CI: –0.06 to 0.58), SEM = 168 s, and MDC95 = 466 s, indicating considerable variability across repeated sessions. Percentage drifts did not significantly differ between sessions for either the min–max (p = 0.52) or min–end (p = 0.22) comparisons. In contrast, the absolute rate of change from minimum to maximum was higher in session 2 (0.62 ± 0.50 vs. 0.50 ± 0.45 mmol·L^−1^·min^−1^; p = 0.004), whereas the minimum-to-end absolute drift did not differ significantly (p = 0.13). For time-normalized [NaCl] equivalent kinetics, repeated-measures ANOVA revealed no significant time × session interaction (*F*_(1,84)_ = 0.14, p = 0.78, η^2^ = 0.002) and no main effect of session (*F*_(1,62)_ = 0.33, p = 0.57, η^2^ = 0.005), indicating similar concentration profiles between sessions. In contrast, a significant main effect of time was observed (*F*_(1,84)_ = 41.4, p < 0.001, η^2^ = 0.40), reflecting systematic changes in sweat [NaCl] equivalent over the course of exercise.

### 3.4 Evolution of sweat [NaCl] equivalent over time

Because no significant effects of side, session, or their interactions with time (time × side, time × session) were observed, all recordings were pooled to provide a global representation of the time-normalized [NaCl] equivalent kinetics. This combined dataset included 64 recordings (16 participants × 2 sessions × 2 armbands), thereby increasing the robustness of the temporal analysis.

**Figure 4:**
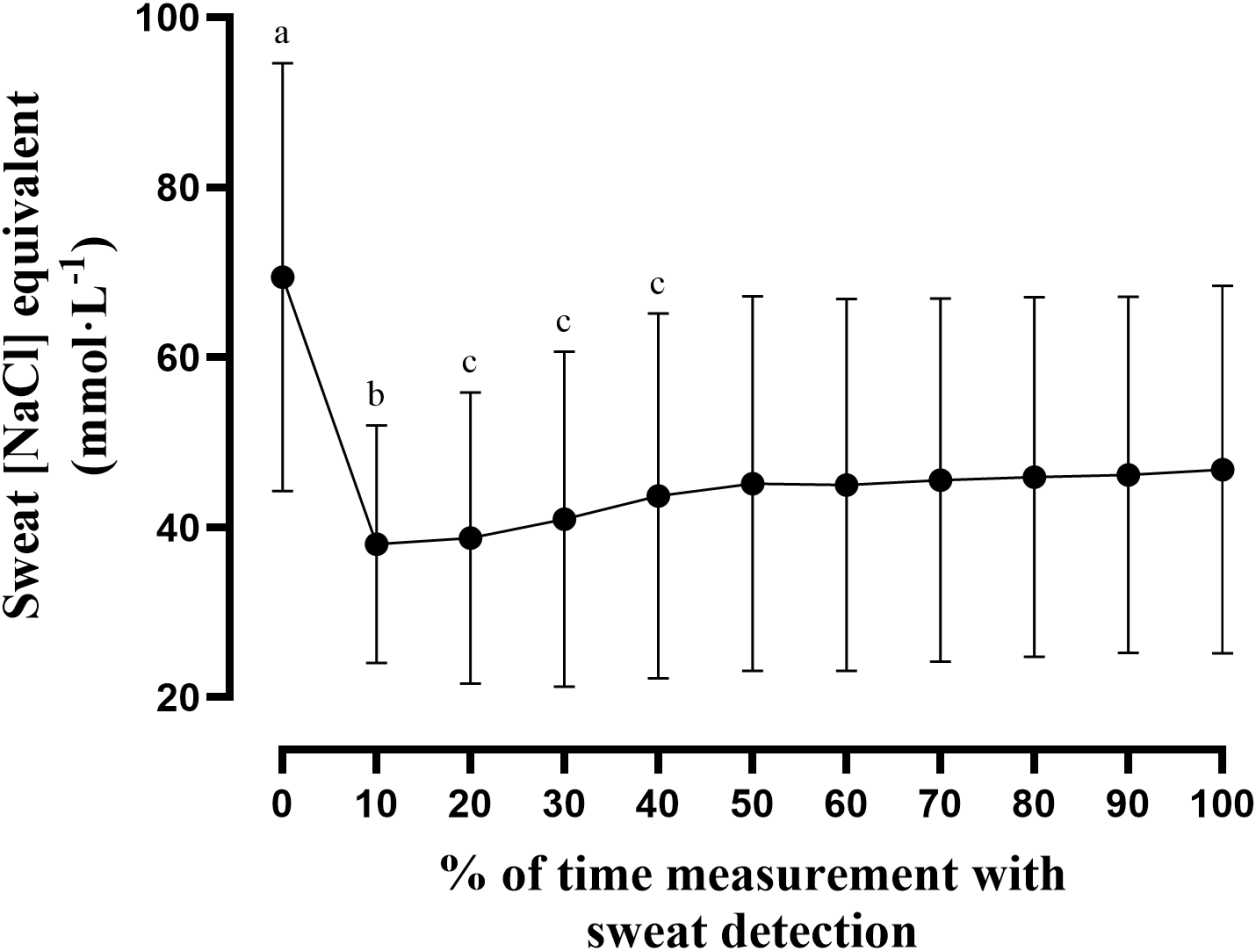
Time-normalized [NaCl] equivalent kinetics during exercise (0–100% measurement duration). At 0% (a), [NaCl] equivalent was significantly higher than all subsequent time points (p < 0.05). At 10% (b), [NaCl] equivalent was significantly lower than values observed at 40–100% and also lower than the initial 0% value (p < 0.05). Between 20% and 40% (c), [NaCl] equivalent values were significantly lower than the 0% value and all subsequent time points (p < 0.05).

**Figure 4** illustrates the time-normalized [NaCl] equivalent kinetics, showing a marked peak at sweat onset (0%), followed by a rapid decline immediately after the peak (10%), then a gradual increase (20–50%) before reaching a stable plateau toward the end of exercise.

## 4 Discussion

The present study aimed to evaluate the validity and reliability of the BeOne armband for continuous monitoring of sweat [NaCl] equivalent during exercise. The main findings were fourfold. First, the armband demonstrated excellent analytical validity for conductivity measurement against standard NaCl solutions, with very low variability and near-perfect agreement across devices. Second, within-session comparisons showed no systematic side effect, as time-normalized [NaCl] equivalent kinetics did not differ between arms, although a minor difference was observed in the absolute drift. Third, between-session reproducibility was limited for sweat onset, yet the overall time-normalized [NaCl] equivalent kinetics, sweat rate, heart rate, and RPE were consistent across sessions. Finally, continuous recordings revealed a distinctive sweat [NaCl] equivalent profile, characterized by an initial peak at sweat onset, followed by a rapid decline, a gradual increase, and a stable plateau toward the end of exercise.

### 4.1 Part A: validity with standard solutions

The BeOne armband provides highly precise and reproducible measurements of solution conductivity, with minimal bias and excellent inter-device agreement when tested against standardized NaCl solutions. These results should be interpreted strictly as a validation of the device’s conductimetric performance, and not as evidence of equivalence with laboratory-based measurements of sweat sodium concentration. Conductivity does not quantify individual ions but reflects the total ionic strength of a solution. As previously reported, this approach leads to systematic differences compared with ion-specific techniques. Boisvert and Candas (1994) reported a statistically significant overestimation of sweat electrolyte concentration by conductivity-based analyzers, with a 95% confidence interval corresponding to an error of ~14% (≈11 mmol·L^−1^), a bias that was particularly pronounced at low electrolyte concentrations measured at sweat onset. Similarly, Goulet et al. (2017) showed that ion conductivity exhibited the greatest disagreement relative to ion chromatography, with the highest coefficient of variation (12.3%) among analytical techniques and concentration-dependent biases ranging from +15.7 mmol·L^−1^ at 20 mmol·L^−1^ to +5.7 mmol·L^−1^ at 110 mmol·L^−1^. In the present study, additional regression analysis of the Bland–Altman differences also revealed a small proportional bias across the calibration range, with slightly higher positive deviations at higher concentrations. However, the absolute magnitude of this bias remained low relative to the tested physiological range. These discrepancies largely reflect matrix effects and the contribution of non-sodium ionic species (e.g., K^+^, lactate, other anions) to total conductivity, which is an inherent limitation of conductimetric sensing compared with ion-selective approaches (Hassan and Fathy, 2024, 2024). Importantly, both studies concluded that, when considered against the normal biological variability of sweat electrolyte concentration (≈ ± 12–15%), the practical implications of this imprecision are generally limited under most exercise conditions. As such, conductivity remains a pragmatic method for field-based applications requiring rapid and continuous measurements. This perspective is supported by recent work on wearable systems such as Flowbio, which also rely on sweat conductivity to estimate sodium concentration. Bandiera et al. (2026) showed that conductivity-based estimates did not match laboratory flame photometry but were comparable to a commonly used field analyzer (e.g. LAQUAtwin-Na-11) and unlikely to meaningfully affect practical sodium replacement recommendations. Collectively, these findings indicate that the BeOne should not be viewed as a laboratory-equivalent sweat sodium analyzer, but rather as a robust tool for continuous conductimetric monitoring of sweat, best suited for relative intra-individual monitoring of temporal changes in sweat electrolyte dynamics rather than absolute quantification. Within current technological and practical constraints, conductivity remains the most feasible approach for continuous field-based assessment of sweat electrolytes.

### 4.2 Part B.1: side effect (right vs. left arm)

For within-session comparisons, sweat onset did not differ between the left and right arms, with moderate reliability as indicated by the ICC. Similarly, most indices of sweat [NaCl] equivalent drift were comparable between arms. Only the absolute rate of change from minimum to maximum was higher on the left arm, suggesting a minor side effect. Importantly, time-normalized [NaCl] equivalent kinetics revealed no differences between arms, indicating that the overall temporal pattern of sweat salt concentration was consistent regardless of measurement side. This agrees with previous studies using absorbent patches, which reported no systematic side differences in forearm sweat [Na^+^] (Baker et al., 2018; Dziedzic et al., 2014).

### 4.3 Part B.2: session effect (test–retest)

Between-session comparisons revealed a different pattern. Although mean sweat onset did not differ across test–retest, reproducibility was low (ICC = 0.29, SEM ≈ 3 min), highlighting substantial day-to-day variability. Practically, this suggests that sweat onset may detect large physiological adaptations (e.g., earlier onset following heat acclimation) (Nadel et al., 1974; Roberts et al., 1977), but changes must exceed the SEM to be meaningful. Most indices of sweat [NaCl] equivalent drift were consistent across sessions, except for a slightly higher absolute min–max rate of change in session 2. This isolated difference is unlikely to reflect a systematic effect and may instead relate to session-specific variability. Finally, time-normalized [NaCl] equivalent kinetics again showed a robust effect of time but no significant session or interaction effect, reinforcing that the general trajectory of sweat [NaCl] equivalent is reproducible across days. Previous work using patch or closed-pouch collection has reported CVs of 8–16% for sweat [Na^+^], indicating that the reproducibility of continuous measures is in a similar range and likely influenced by the collection method (Baker et al., 2009; Hayden et al., 2004).

### 4.4 Evolution of sweat [NaCl] equivalent over time

The continuous recordings revealed a consistent temporal pattern, characterized by a sharp peak at sweat onset, followed by a rapid decline, a gradual increase, and a stable plateau toward the end of exercise. Importantly, no comparable peak was observed when standardized NaCl solutions were directly introduced into the device during Part A, suggesting that this feature is unlikely to result from an intrinsic sensor response, electrode polarization, or microfluidic artifact. Similar early elevations in mineral concentrations have been reported for calcium, magnesium, zinc, and copper when sweat was collected without proper skin preparation, reflecting contamination from ions trapped in sweat pores or the epidermis (Ely et al., 2011; Montain et al., 2007). Although such effects have not been described for [Na^+^], the systematic appearance of the peak in the present study may reflect a similar mechanism. Despite rinsing and wiping the skin with deionized water before armband placement, residual NaCl equivalent may transiently elevate the initial readings. Alternatively, this initial peak may also relate to early sweat gland secretory mechanisms, as activation of the Na^+^–K^+^–Cl^−^ cotransporter (NKCC1) promotes osmotic water movement into the gland lumen to initiate sweat secretion (Cui and Schlessinger, 2015), potentially resulting in a transiently higher electrolyte concentration in the initial sweat. From a practical perspective, values recorded after the peak may better reflect the steady-state composition relevant to hydration strategies.

Several studies have reported that higher sweat rates are accompanied by increased sweat [Na^+^] concentrations, suggesting a relationship between the two variables (Buono et al., 2007; Dziedzic et al., 2014). Buono et al. (2007) hypothesized that this association results from proportionally reduced sodium reabsorption in the sweat duct when sweat is secreted more rapidly. Thus, the progressive rise in sweat [NaCl] equivalent may represent the balance between increased secretion and limited reabsorption as sweat production accelerates to support thermoregulation (Buono et al., 2008).

### 4.5 Limitation

A primary limitation is that the BeOne was not compared against a laboratory gold-standard method specifically quantifying sweat sodium concentration. Likewise, no time-matched local sweat sampling was performed in the immediate vicinity of the device to allow comparison between the conductivity-derived signal and an averaged ion-specific measurement over a defined exercise interval; this should be considered an important step for future validation studies. Accordingly, the present findings should not be interpreted as a physiological validation of sweat sodium concentration, but rather as a validation of the conductimetric signal and its reproducibility during exercise. In the present design, calibration solutions were instead validated against a reference conductivity analyzer (Sweat Check), which is consistent with the sensing principle of the device. Conductivity-based measurements are not equivalent to ion-specific laboratory analyses, and their limitations in sweat have been well documented (Boisvert and Candas, 1994; Goulet et al., 2017). More specifically, the potential contribution of other ionic species and matrix effects inherent to human sweat was not directly quantified in the present study. Furthermore, local sweat rate was not measured simultaneously, which limits the interpretation of the respective contributions of sweat flow dynamics and electrolyte composition to the temporal signal recorded by the device. Nevertheless, this approach has long been used in clinical practice (e.g., cystic fibrosis screening) and is considered sufficiently accurate for applied sport-science purposes, such as hydration assessment, athlete classification (~25 mmol·L^−1^; Montain et al., 2006), and heat acclimation monitoring (decreases by ~30–50%; Allan and Wilson, 1971; Buono et al., 2018; Tyler et al., 2016). While laboratory analyses remain necessary for high-precision research, their logistical constraints limit field applicability. Within applied exercise physiology, where the emphasis is on temporal dynamics and intra-individual tracking, conductivity-based monitoring appears justified, provided its analytical limitations are clearly acknowledged.

## 5 Conclusion

This study provides the first comprehensive evaluation of the BeOne armband for continuous monitoring of sweat conductivity, combining validation against reference solutions, within-session comparisons, and a test–retest design. The ability to capture uninterrupted sweat [NaCl] equivalent kinetics during exercise represents a major strength, offering novel insights into the temporal patterns of conductivity-derived sweat electrolyte signals. Future research should further investigate the applicability and physiological relevance of these novel continuous sweat conductivity-derived indicators across different populations (training status, age, sex), environmental conditions (heat stress), and exercise intensities, and more ecologically valid field settings including fluid intake during exercise. In addition, direct comparisons with other commercially available wearable systems and alternative sensing strategies (Baker et al., 2022a; Bandiera et al., 2026), including potentiometric and hybrid electrochemical approaches (Hassan and Fathy, 2026), would help better position the BeOne within the current landscape of real-time sweat monitoring technologies. Simultaneous assessment of metabolic heat production and body temperatures would further clarify the links between thermoregulatory demands, sweat rate, and sweat [NaCl] kinetics, ultimately improving hydration monitoring and the individualization of fluid replacement strategies in athletes.

## Data Availability

The raw data supporting the conclusions of this article will be made available by the authors, without undue reservation.
